# Three-Dimensional Macronutrient-Associated Fos Expression Patterns in the Mouse Brainstem

**DOI:** 10.1371/journal.pone.0008974

**Published:** 2010-02-01

**Authors:** Jessica Schwarz, Jasmine Burguet, Olivier Rampin, Gilles Fromentin, Philippe Andrey, Daniel Tomé, Yves Maurin, Nicolas Darcel

**Affiliations:** 1 AgroParisTech, CRNH-IdF, UMR914 Nutrition Physiology and Ingestive Behavior, Paris, France; 2 INRA, CRNH-IdF, UMR914 Nutrition Physiology and Ingestive Behavior, Paris, France; 3 INRA, UMR 1197 Neurobiologie de l'Olfaction et de la Prise Alimentaire, Domaine de Vilvert, Jouy-en-Josas, France; 4 Université Paris-Sud 11, UMR 1197, Orsay, France; 5 IFR 144 Neuro-Sud Paris, Paris, France; 6 UPMC Univ. Paris 06, Paris, France; University of Camerino, Italy

## Abstract

**Background:**

The caudal brainstem plays an important role in short-term satiation and in the control of meal termination. Meal-related stimuli sensed by the gastrointestinal (GI) tract are transmitted to the area postrema (AP) via the bloodstream, or to the nucleus tractus solitarii (NTS) via the vagus nerve. Little is known about the encoding of macronutrient-specific signals in the caudal brainstem. We hypothesized that sucrose and casein peptone activate spatially distinct sub-populations of NTS neurons and thus characterized the latter using statistical three-dimensional modeling.

**Methodology/Principal Findings:**

Using immunolabeling of the proto-oncogene Fos as a marker of neuronal activity, in combination with a statistical three-dimensional modeling approach, we have shown that NTS neurons activated by sucrose or peptone gavage occupy distinct, although partially overlapping, positions. Specifically, when compared to their homologues in peptone-treated mice, three-dimensional models calculated from neuronal density maps following sucrose gavage showed that Fos-positive neurons occupy a more lateral position at the rostral end of the NTS, and a more dorsal position at the caudal end.

**Conclusion/Significance:**

To our knowledge, this is the first time that subpopulations of NTS neurons have be distinguished according to the spatial organization of their functional response. Such neuronal activity patterns may be of particular relevance to understanding the mechanisms that support the central encoding of signals related to the presence of macronutrients in the GI tract during digestion. Finally, this finding also illustrates the usefulness of statistical three-dimensional modeling to functional neuroanatomical studies.

## Introduction

The central nervous system (CNS) controls food intake integrating a broad variety of both environmental and internal inputs [1]. The caudal brainstem, which is the main recipient of information from the GI tract, is a key region in this control. Particularly during digestion, a complex signaling cascade informs brainstem centers of the nature and quantity of macronutrients entering the organism [1], [2]. Both non-specific mechanical and macronutrient-specific meal-related stimuli are sensed in the GI tract, where they induce the release of gut peptides. Gut peptides and absorbed nutrients act either on the sensory vagal afferents that innervate the gut and the hepato-portal area, or directly in the brain through the bloodstream [3]–[8]. Within the central nervous system, vagal afferents synapse in the *nucleus tractus solitarii* (NTS) located in the caudal brainstem. In addition, the NTS receives input from the area postrema (AP), which displays elevated permeability of the blood-brain barrier and is sensitive to the blood-borne signals linked to ingested macronutrients [9].

Nutrient-specific behavioral responses have been observed, suggesting that the brain receives and integrates nutrient-specific signals [10]. Very little is known about the central encoding modalities of macronutrient-specific detection during digestion [11], [12]. This information would be of considerable value to a clearer understanding of the physiological mechanisms that control food intake and its dysfunctions. All macronutrients exert different impacts on food intake control, especially regarding the degree of satiety. There is evidence to suggest that different macronutrients generate specific signals during their absorption in the gastrointestinal tract. For instance, cholecystokinin (CCK) is released in response to the presence of dietary fat and protein in the gut lumen, while serotonin (5-HT) levels rise in response to the presence of carbohydrates and lipids [7], [10], [13]–[15]. Furthermore, by quantifying the proto-oncogene Fos as a marker of neuronal activity, it has been shown that different macronutrients are associated with different levels of activation in the NTS and AP [12]. This effect is positively related to their potency to inhibit food intake [11]. According to Phifer et al., activation of the caudal brainstem in correlation with macronutrient detection is dependent on the type of nutrient rather than its caloric value [11].

Very few studies have investigated the central encoding modalities of macronutrient-associated signals, and more precisely whether patterns of neuronal activity within the brainstem are specific to macronutrients. We therefore assessed the effects of different macronutrients on Fos expression and investigated whether specific activation patterns could be detected. Following the administration of sucrose or casein peptone, we quantified Fos expression throughout the rostrocaudal extent of the NTS. We compared: a) the total number of Fos-expressing neurons in the brainstem, AP and NTS, b) their distribution along the rostrocaudal axis, and c) the spatial distribution of NTS neurons using three-dimensional reconstructions [16] and statistical mapping of neuronal population densities [17]. Our findings indicate that macronutrients in the gastrointestinal tract elicit distinct patterns of neuronal activity in the caudal brainstem.

## Materials and Methods

### Animals

Male C57BL/6J mice (Harlan Laboratories, Inc., Horst, The Netherlands) aged 6 to 8 weeks at the time of the experiment were housed in groups of 4 to 6 at 22±1°C under a 12-hour light/dark cycle (lights off at 20.00 h). The mice were left for one week to recover from transport and become habituated to their housing conditions. All experiments were carried out according to the guidelines of the French National Committee for Animal Care and the European Convention of Vertebrate Animals Used for Experimentation, under European Council Directive 86/609/EEC dated November, 1986.

### Macronutrient Administration

For one week prior to the experiment, mice were gavaged daily with increasing volumes of water in order to accustom them to the experimental procedure. On the day of the experiment, following an 18-hour fasting period, and at the beginning of the light phase (08.00 h), mice were gavaged with an iso-caloric (1.2 kcal), iso-volumetric (700 µl) and iso-osmotic load, using a 20G/30 mm-long feeding needle (Fine Science Tools GmbH, Heidelberg, Germany). Animals in the treatment group received either carbohydrate (0.4 g/ml D(+)-Sucrose; AnalaR NORMAPUR®, VWR International, Strasbourg, France) or protein (0.4 g/ml Peptone from casein, enzymatic digest; Fluka, Sigma-Aldrich, St Quentin Fallavier, France) diluted in water. Two control groups were used, one receiving 700 µl of water as a control for gastric distension and the other not being subjected to any stimulation. Three mice were processed in each experimental group.

### Brain Fixation and Collection

Ninety minutes after administration of the loads, the mice received a lethal i.p. injection of sodium pentobarbital (4 ml/kg CEVA SANTE ANIMALE, Centravet, Plancoët, France) and were subsequently transcardially perfused via a 26G needle placed in the left cardiac ventricle with 50 ml of 0.9% saline (NaCl, VWR International) supplemented with 2% NaNO_2_ (pH 7.5, 3 min, Sigma-Aldrich), followed by 100 ml of 4% paraformaldehyde (Merck KGaA, VWR International). Their brains were removed and transferred to a 4% formaldehyde fixative solution for 4–6 hours, and then placed overnight at 4°C in 0.1 M phosphate buffer containing 15% sucrose. The brains were then frozen in isopentane (Sigma-Aldrich) at −35+/−3°C, embedded in Tissue-Tek® (Tissue-Tek® O.C.T. Compound, Sakura Finetek Europe B.V., Zoeterwoude, The Netherlands) and stored at −20°C until sectioning. Approximately 100 coronal 20 µm-thick brainstem sections, covering the entire rostrocaudal extent of the NTS, were cut using a cryostat (CM3000, Leica, Germany) at −19°C. Brain positions were localized using a stereotaxic atlas [18]. Odd- and even-numbered sections were mounted on two separate sets of gelatin-coated glass slides and stored at −80°C until further processing.

### Fos Immunohistochemistry

Immunostaining of the Fos protein was performed on the odd-numbered sections at room temperature as follows: a) rinsing in 0.01 M sodium phosphate buffer containing 0.9% NaCl (PBS, 10 min, pH 7.4), b) incubation in 0.5% Triton X-100 (Sigma-Aldrich) and 2% BSA (Sigma-Aldrich) in PBS for 1 h, c) overnight incubation with Fos primary antibody (Anti-c-Fos (Ab-5) (4–17) Rabbit pAB, Calbiochem, VWR International) at a dilution of 1∶5000 in PBS supplemented with 0.5% Triton X-100 and 2% BSA, d) 3 rinses (10 min each) in 0.1% skimmed milk (Régilait, St-Martin-Belle-Roche, France) in PBS, e) 3 h incubation with the biotinylated secondary antibody (biotinylated anti-rabbit IgG (H+L), Vector Laboratories, AbCys S.A., Paris, France) diluted 1∶200 in 0.5% Triton X-100 and 2% BSA in PBS, f) 3 rinses in PBS, g) 30 min incubation in 30% H_2_O_2_ (Merck KGaA, VWR International) in PBS h) 3 rinses in PBS, i) 30 min treatment with avidin-biotin complex (Vectastain Elite ABC kit, Vector Laboratories), j) 2 washes in PBS, k) 1 rinse in Trizma solution (pH = 7.6, Trizma® HCl and Trizma® Base, Sigma-Aldrich), l) revelation of peroxidase activity using 0.5% diaminobenzidine terahydrochloride (DAB, Sigma-Aldrich) with 50 ppm of 30% H_2_O_2_ for 8–10 min, m) DAB reaction stopped in Trizma solution, n) 3 rinses in water (10 min each), o) dehydration in ethanol (VWR International) rinses, immersion in graded toluene (VWR International) and cover slipping using DePeX mounting medium (VWR International). A Fos-positive control and a negative control, obtained by omitting the primary antibody on a sample of two sections from each animal, were processed during each experiment to check for antibody specificity.

### Luxol Fast Blue/Neutral Red Histochemistry

Luxol fast blue/neutral red histochemistry was carried out on even-numbered sections as follows: a) 3 min rinse in water and 3 min in 95% ethanol, b) 4 h incubation at 58°C in 1% Luxol fast blue solution (1% Solvent Blue 38, 95% ethanol, 0.5% glacial acetic acid, Sigma-Aldrich), c) rinse in water (3 dips), d) differentiation in 0.1% Li_2_CO_3_ (Sigma-Aldrich) for 2–3 min, e) wash in water (3 dips), f) counterstaining for 10 min in 0.5% neutral red (pH 4.5, Neutral Red dye (Sigma-Aldrich) in water), g) 2 rinses in water, h) dehydration by graded ethanol rinses, immersion in graded toluene and cover-slipping with DePeX mounting medium (VWR International).

### Image Digitization

**Figure 1:**
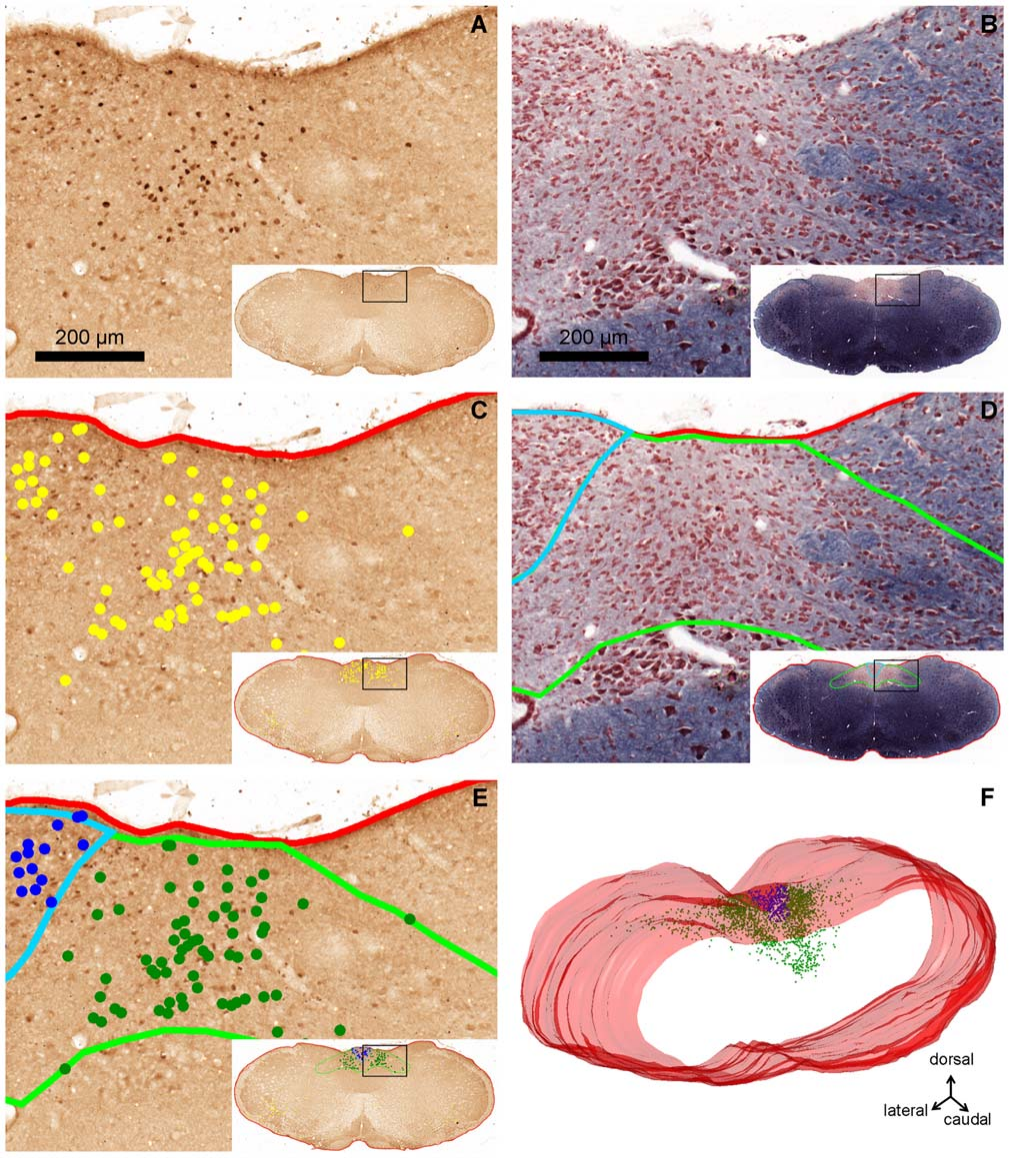
Reconstruction of a three-dimensional model using serial sections of mouse brainstem. A, C, E, coronal section immunolabeled for nuclear Fos protein. B, D, adjacent section with Luxol fast blue/neutral red counterstaining. A, brown spots are evidence for Fos-positive neurons in the dorsomedial part of the brainstem. Insert: whole brainstem section, the black rectangle indicating the enlarged picture. B, myelinated fibers labeled in blue and neuronal cell bodies labeled in red. C, section identical to A, the red line delineating the brainstem contour and yellow spots labeling Fos-positive neurons. D, section identical to B, with delineated brainstem (red line), NTS (light green) and the AP contour (light blue). E, section identical to A, NTS and AP contours from D are appropriately superimposed on C, allowing the assignment of labeled neurons (C) to the NTS (dark green) or AP (dark blue). F, the stack of brainstem contours (red) and labeled neurons (dark blue, dark green) generate a three-dimensional model, showing the caudal part of the brain.

For image acquisition *Mosaic* software (Explora Nova, La Rochelle, France) was used. This application, installed on a PC equipped with a digitizer board (Matrox Meteor2/MC4, Dorval, QC, Canada), used a Ludl-MAC5000 controller to drive a motorized stage (Marzhauser, Wetzlar, Germany) fitted to a microscope (AxioPlan2 Imaging, Carl Zeiss, Germany) equipped with a 3CCD camera (Hitachi HV-C20A, Tokyo, Japan). This setting enabled the acquisition of large surface areas as mosaics of images at a resolution appropriate to the identification of structures of interest. Two image stacks were acquired for each animal. Odd-numbered sections (Fos immunostaining) were digitized using a x20 lens (pixel resolution: 0.41 µm) to enable the identification of Fos-positive neurons (Figure 1A). Even-numbered sections (Luxol fast blue staining) were digitized using an x10 lens (pixel resolution: 0.82 µm), which was sufficient to identify the NTS and AP regions (Figure 1B).

### Three-Dimensional Model Construction

In all mice, the same rostrocaudal region of the brainstem (from Bregma −8.3 mm to Bregma −6.2 mm according to a stereotaxic atlas [18]) was sampled in coronal sections. Three-dimensional models of the brainstem and cell populations were generated using *Free-D*, a reconstruction and modeling software [16] (Figure 1C–F). For this purpose, in each mouse, structures of interest identified on the digitized images were manually segmented: the brainstem contour and Fos positive cells on Fos-labeled sections (Figure 1C), the brainstem contour and those of the NTS and AP on Luxol-stained sections (Figure 1D). In a second step, the brainstem contours of both series of sections were registered using affine transformations: brainstem contours from Luxol-stained images were registered on a section-by-section basis onto the contours of Fos-immunolabeled sections. The same transformations were applied to NTS and AP contours in order to correctly superimpose them on the Fos images (Figure 1E). Finally, consecutive images of the Fos series were manually registered to yield a three-dimensional model of the brainstem encompassing the two scatters of points corresponding to NTS- and AP-Fos-positive cells (Figure 1F).

### Spatial Normalization of Three-Dimensional Models

Statistical representations of Fos-positive neuron positions were built according to the following steps: a) spatial normalization of the individual three-dimensional models, b) computation of one statistical NTS neuron density map per experimental group, c) extraction of isodensity surfaces. All steps have been described elsewhere [17], [19] and are only briefly summarized here. Spatial normalization requires a reference structure which, in the present experiments, was the brainstem envelope. This was performed as follows: brainstem models were registered and averaged [19], [20]. Polynomial deformations, mapping each individual model onto the average model, were then computed and propagated to NTS Fos-positive cell positions. This procedure generated spatially normalized three-dimensional models which could be superimposed to visualize the three-dimensional organization of NTS Fos-positive cells (see Figure S1). Morphological variability was assessed by computing the point-to-point root mean square distance between individual brainstem envelopes and the averaged model.

### Data Analysis

#### Estimated total number of Fos-positive neurons

The numbers of Fos-positive neurons were determined in the regions of interest. The Abercrombie correction factor [21] was applied to correct for duplicate counting. The numbers of Fos-positive neurons in the three experimental groups were compared using the Kruskal-Wallis rank sum test in the R statistical software package (version 2.8.1) [22]. In brain regions where a significant difference was found, pair-wise comparisons were performed using the Wilcoxon rank sum test.

#### Rostrocaudal distribution of Fos-positive neurons

Fos-positive cells in each mouse were sorted according to their coordinates along the rostrocaudal axis, and the probability density function was assessed using Gaussian kernel density estimations [23]. For each animal, the individual probability density curve of rostrocaudal Fos-positive neuron positions was multiplied by the estimated total number of Fos-positive neurons and the resulting curves were averaged within each experimental group to yield an estimate of the rostrocaudal distribution of neurons. For raw data and density estimation curves, see Figure S2.

#### Three-dimensional distribution of Fos-positive neurons in the NTS

In each group, the local density of Fos-positive neurons was statistically estimated at each node of the grid from all NTS neuron sets of the group [17]. To achieve this, a three-dimensional grid overlapping all normalized NTS Fos-positive neuron positions over all groups was defined. Therefore, each group was processed separately as described below. The local density of Fos-positive neurons was then estimated statistically at each node of the grid from all NTS neuron sets for each group. The resulting three-dimensional grid of local density estimates constituted the density map of a group. To enable visual analysis and interpretation, three-dimensional isodensity surfaces were computed from these density maps. The isodensity threshold was chosen so that the corresponding iso-surface encompassed 90% of Fos-positive neurons in the group.

## Results

### Total Number of Fos-Positive Neurons

**Figure 2:**
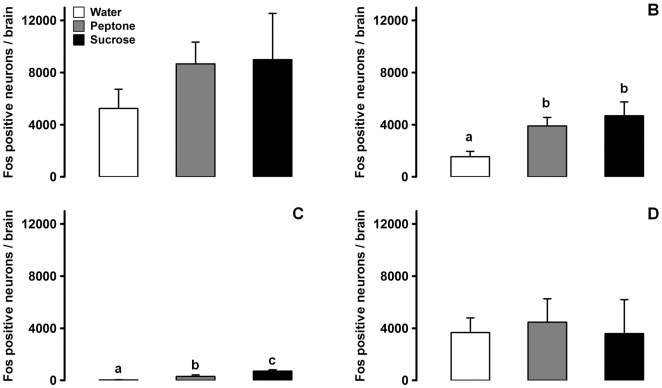
Number of Fos-positive neurons in the brainstem of mice following gavage with different loads. A, total number of Fos-positive neurons in the entire brainstem after gavage with casein peptone (grey bars), sucrose (black bars) or water (white bars). No differences were found between these groups (*p* = 0.6703). B, number of Fos-positive neurons in the NTS. The water group revealed a significantly lower number of labeled cells than the other two groups (p<0.05 for both comparisons). C, number of neurons in the AP. The number of Fos-positive cells was treatment-dependent (*p* = 0.0273). All pair-wise comparisons were statistically significant (p<0.05). D, number of neurons outside the NTS and AP. No statistical difference was found (*p* = 0.7326). Data are presented as mean ± SEM after Abercrombie correction. Bars labeled with different letters differ according to the Wilcoxon rank sum test, p<0.05.

No Fos-positive neurons were observed on brainstem sections from control mice that did not receive intragastric loads (data not shown). By contrast, Fos-positive neurons were present in sections from all other experimental groups, as illustrated in Figure 2 which shows the number of Fos-positive neurons in the brainstem of mice after water, peptone or sucrose gavage. When considering the whole brainstem (Figure 2A), no difference was found between the three experimental groups. In the NTS (Figure 2B), the water group displayed a significantly smaller number of Fos-positive neurons than the two others, while no difference was found between the peptone and sucrose groups. In the AP (Figure 2C), the number of Fos-positive neurons was nutrient-dependent and all pair-wise comparisons reached statistical significance (water < peptone < sucrose). Outside the NTS and AP (Figure 2D), no differences were observed between groups.

### Rostrocaudal Distribution of Fos-Positive Neurons

**Figure 3:**
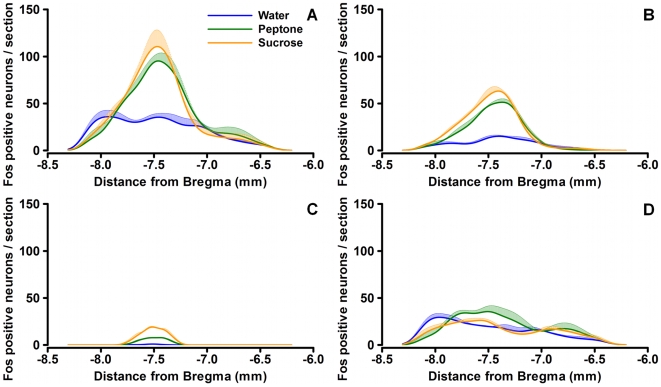
Distribution of Fos-positive neurons along the rostrocaudal brain axis. Smoothed histograms of the number of Fos-positive neurons in the entire brainstem (A), the NTS (B), the AP (C) and the entire brainstem without an object count from NTS and AP (D). Data are expressed as mean ± SEM. The X-axis represents the distance in millimeters from Bregma.

Figure 3 represents the rostrocaudal distribution of Fos-positive neurons in the whole brainstem (Figure 3A), the NTS (Figure 3B) and the AP (Figure 3C). Figure 3D shows their distribution outside these latter structures, where Fos immunoreactive neurons displayed a relatively uniform distribution with no marked differences between the water, sucrose and peptone groups. In the whole brainstem (Figure 3A), the distribution of Fos-positive neurons following water gavage was similar, with a maximum at around Bregma −8 mm and a slow decrease in the rostral direction. In the NTS (Figure 3B) and AP (Figure 3C), water gavage induced a weak Fos response. Thus, in the brainstem, Fos immunoreactive neurons in response to water gavage were mostly localized outside the NTS and AP. The picture was different concerning the responses to sucrose and peptone, which in the brainstem (Figure 3A) induced a peak of Fos immunoreactive neurons centered around Bregma – 7.5 mm. This peak was due to Fos immunoreactive neurons in the NTS and AP, with a major contribution from the NTS (see ordinate scales in 3B and 3C). Within the NTS, more Fos immunoreactive neurons were located in the NTS section located at the level of the AP (Bregma −7.2 mm to −7.8 mm).

### Three-Dimensional Distribution of Fos Immunoreactive Neurons in the NTS

**Figure 4:**
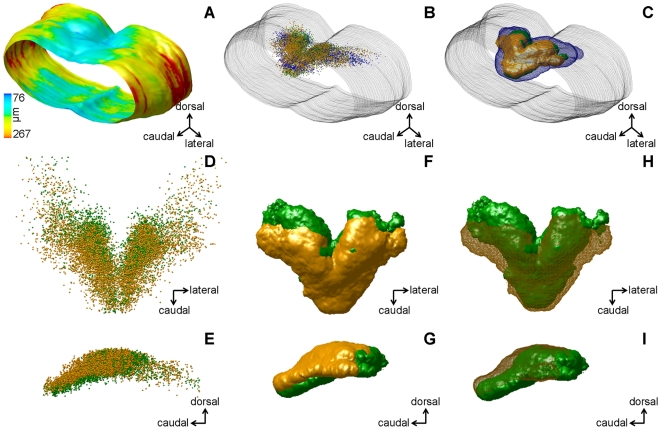
Merged normalized three-dimensional models and statistical distributions of cell populations. A, caudal view of the averaged brainstem envelope from 8 mice. The color code (bottom left) reflects the positional variability of each point of the model, from the least variable (blue) to the most variable (red). Variability is calculated as the root mean square distance (in µm) between a given point on the average model and homologous points on the individual models. Color coding for the following images: models for the water group are presented in blue, for the protein group in green and for the sucrose group in orange. B, all Fos-positive NTS neurons from the 8 mice displayed after normalizing their position according to the averaged brainstem, represented in grey mesh mode. Same orientation as A. C, superimposition of three isodensity surfaces, each corresponding to NTS Fos-positive neurons from one experimental group. Each iso-surface includes 90% of Fos-positive neurons from the experimental group. Same orientation as A. D, all NTS Fos-positive neurons in the peptone and sucrose groups are represented in a dorsal view, after spatial normalization. E, same as D, lateral view. F and G, dorsal and lateral views of isodensity surfaces for the peptone and sucrose groups. Thresholds were selected so that the isodensity surfaces encompass 90% of Fos-positive neurons in each group. H and I, same as F and G, respectively, but the isodensity surface of the sucrose group is displayed in mesh mode. Models in A, B and C, as well as in D, E, F, G, H, and I are displayed at the same magnification.

Figure 4A represents the average brainstem model calculated after spatial registration of the individual three-dimensional brainstem models obtained from eight mice (n = 3 for water- and sucrose-loaded groups, n = 2 for the peptone-loaded group). Note that in one peptone loaded mouse, the brainstem model was incomplete and therefore this animal was excluded from further analyses. The average three-dimensional model includes a color code expressing variability which was greater in the lateral parts of the brainstem than in the dorsal and ventral parts.

Superimposition of all eight spatially normalized NTS cell populations (Figure 4B) did not enable the identification of differences between neuronal sets activated by the three treatments. In contrast, a representation of these neuronal sets by isodensity surfaces computed from statistical density maps (Figure 4C) allowed a qualitative visual analysis. The surface corresponding to the water-loaded group encompassed the two other surfaces (see also Film S1).

Figures 4D to 4I focus on data from the peptone and sucrose groups. Superimposition of the populations of Fos-positive neurons in these groups (Figures 4D and 4E), showed that, as a whole, they had similar rostrocaudal, mediolateral and dorsoventral extensions. However, the respective isodensity surfaces were different (Figures 4F–4I). Caudally, peptone- and sucrose-induced Fos immunoreactive neurons reached the same position, while rostrally, the peptone group had a greater extension. Dorsoventrally, neurons from the peptone-treated group extended more ventrally while neurons from the sucrose-treated group occupied a more dorsal position. Finally, neurons from the peptone group occupied a narrower volume along the mediolateral axis. In order to assess the degree of overlap between the two populations, we determined their total volume and estimated the portion of this volume that was simultaneously occupied by the two groups. This intersection occupied 53% of the united volume.

## Discussion

This study shows that specific neuronal activity patterns are induced in the NTS by various macronutrients and that these different patterns can be revealed by three-dimensional density maps. These results are in favor of the processing of specific gastrointestinal information by subpopulations of NTS neurons. This conclusion represents an important contribution to the understanding of the mechanisms involved in macronutrient signaling to the brain.

The NTS is the point of convergence of peripheral information generated by the GI tract during digestion. It integrates nervous signals from vagal afferents and from central AP neurons [24]. It has been known for some time that in the rat, the NTS is organized in a viscerotropic manner [2]. In this scheme, NTS neurons receiving information from the upper GI tract (i.e. esophagus, stomach and duodenum) are located in the vicinity of the AP. For other sensory modalities such as audition, olfaction or touch, functional organizations have been revealed by three-dimensional analysis [25]-[28]. Functional activity maps may also exist for the NTS with respect to visceral sensitivity. Although it has been claimed that no functional mapping exists in the NTS [11], no spatial analyses have been performed in this region. We therefore undertook a three-dimensional study of neuronal activity following the administration of macronutrients. To take account of both experimental and inter-individual variability, a statistical strategy was adopted. We took advantage of a recently reported method for spatial normalization and statistical three-dimensional modeling of neuronal populations [17] in order to address the question of a possible functional segregation of NTS neurons.

### Macronutrient-Elicited Fos Expression in the Brainstem Was Restricted to NTS and AP Neurons

In both the peptone and sucrose groups, the major contribution of total Fos immunoreactive neurons in the brainstem came from the NTS, with a minor contribution from the AP. The contingent of water-induced Fos-positive neurons was mainly located outside the NTS and AP, and was very limited in these two regions, suggesting that the Fos labeling induced by sucrose and peptone in the NTS and AP was stimulus-specific. Since no Fos immunoreactive neurons were observed when no load was applied, the Fos immunoreactivity observed outside the NTS and AP probably resulted from gastric distension during gavage. Taken together, these results are in line with the findings of previous reports [11], [12], [29] which also showed that macronutrients administered in the GI tract elicited specific Fos responses in the NTS and to a lesser extent in AP, and that gastric distension induced Fos expression at the same positions, independently of the macronutrient type.

### Within the NTS, Macronutrients Elicited Specific Patterns of Neuronal Activity

A functional segregation of NTS neuron subpopulations could only be demonstrated after three-dimensional analysis, while in line with previous reports, analysis of the distribution of Fos-positive neurons either on coronal sections or along the rostrocaudal axis showed no difference. Individual three-dimensional models (Figure 1F) provided a clearer view of their spatial organization but were not appropriate for comparative analysis. Model normalization and fusion yielded representations of whole experimental groups (Figure 4D and 4E). These fusion models were the superposition of normalized populations. The larger the experimental group, the bigger was the apparent size of the population and the more confused the representation. Therefore, although synthetic, the representations in Figures 4D and 4E were difficult to analyze visually. Only a computation of density maps made it possible to draw some clearer conclusions. Using this approach, we revealed that two different macronutrients specifically activated two functional subpopulations of NTS neurons. Visual examination of the iso-surfaces extracted from density maps showed a subtle but clear segregation of these neuron sets in the mediolateral, rostrocaudal and dorsoventral directions. However, calculation of their overlap indicated that only one half of their total volume was shared by both subpopulations. Further work is necessary to design and implement statistical tests that will be applicable to three-dimensional data and allow us to confirm our visual conclusions.

### Possible Physiological Bases

Both the rostrocaudal distribution and three-dimensional organization of Fos immunoreactivity showed that activated neurons in the NTS were located in the vicinity of the AP, a finding consistent with the viscerotropic organization previously reported [2]. The segregation between subpopulations of NTS neurons following peptone and sucrose gavage might result from anatomical differences in the gastrointestinal sites of signaling, thus having anatomical rather than functional origins. Moreover, the signaling sites of peptone and sucrose in the intestine are not known sufficiently accurately to suggest a correlation with the observed segregation in the NTS. Considering the greater ability of protein, rather than carbohydrate, to delay gastric emptying [30], [31] one might propose that the observed differences in Fos-positive neuron positions were due to differences in gastrointestinal and absorptive kinetics. Indeed, these alterations certainly modulate the contributions of the signaling sites involved in gut-brain communication during digestion. Independently of the actual origin of these changes, it is clear that with respect to the greater potency of protein than carbohydrate in triggering control of ingestion, and considering the fact that the NTS is the key region in the satiation process, it is legitimate to hypothesize that the segregation of neuronal positions within the NTS reflects central satiation mapping.

### Conclusion and Perspectives

We have thus shown that different macronutrients activate distinct subpopulations of NTS neurons. Because of its complexity, this segregation could only be unmasked using a statistical, three-dimensional approach. Its physiological meaning still requires further investigation, but it is likely to be relevant to understanding how the brain traduces different metabolic situations and their influence on the control of food intake.

The currently proposed conceptual framework of eating behavior studies states that a small subset of brain regions integrates internal and external sensory cues and generates hunger, satiety and satiation. How these brain areas integrate feeding-related information remains largely unknown, mostly because these signals are multiple and signal continuously to the brain. Instead of focusing separately on each factor and its signaling to the brain (e.g., gastric volume or CCK), the method developed here is designed to gain insight into the neurobiology of eating using an integrated approach that focuses on a central representation of a complex internal situation rather than fine, but reduced, action/reaction effects.

## Supporting Information

Figure S1 Three-dimensional models of the brainstem and Fos-expressing neurons. A, fusion of the average brainstem model with single brainstem models for mice gavaged with water. B, fusion of the average brainstem model with single brainstem models for mice gavaged with saccharose. C, fusion of the average brainstem model with single brainstem models for mice gavaged with peptone. D, average brainstem model with the total number of neurons marked in mice gavaged with peptone, E, average brainstem model with the total number of neurons marked in mice gavaged with saccharose, F, average brainstem model with the total number of neurons marked in mice gavaged with water.
(1.69 MB TIF)

Figure S2 Raw data, means and density curves presenting the distribution of Fos-expressing neurons along the rostrocaudal axis.
(0.93 MB TIF)

Film S1 Three-dimensional models of Fos-expressing neurons in the NTS for water (blue) peptone (green) and sucrose (orange)-loaded mice. Start of the film: view from caudal position, turning to a ventral view, then a rostral view, a dorsal view and then back to the caudal view. Second part: rotation around the dorso-ventral axis.
(6.07 MB MOV)

## References

[1] Schwartz MW, Woods SC, Porte D, Seeley RJ, Baskin DG (2000). Central nervous system control of food intake.. Nature.

[2] Norgren R, Smith GP (1988). Central distribution of subdiaphragmatic vagal branches in the rat.. J Comp Neurol.

[3] Raybould HE (1999). Nutrient tasting and signaling mechanisms in the gut. I. Sensing of lipid by the intestinal mucosa.. Am J Physiol.

[4] Raybould HE, Glatzle J, Freeman SL, Whited K, Darcel N (2006). Detection of macronutrients in the intestinal wall.. Auton Neurosci.

[5] Berthoud HR (2004). Anatomy and function of sensory hepatic nerves.. Anat Rec A Discov Mol Cell Evol Biol.

[6] Moran TH, Kinzig KP (2004). Gastrointestinal satiety signals II. Cholecystokinin.. Am J Physiol Gastrointest Liver Physiol.

[7] Raybould HE, Glatzle J, Robin C, Meyer JH, Phan T (2003). Expression of 5-HT3 receptors by extrinsic duodenal afferents contribute to intestinal inhibition of gastric emptying.. Am J Physiol Gastrointest Liver Physiol.

[8] Wei Y, Mojsov S (1995). Tissue-specific expression of the human receptor for glucagon-like peptide-I: brain, heart and pancreatic forms have the same deduced amino acid sequences.. FEBS Lett.

[9] Maolood N, Meister B (2009). Protein components of the blood-brain barrier (BBB) in the brainstem area postrema-nucleus tractus solitarius region.. J Chem Neuroanat.

[10] Tome D (2004). Protein, amino acids and the control of food intake.. Br J Nutr.

[11] Phifer CB, Berthoud HR (1998). Duodenal nutrient infusions differentially affect sham feeding and Fos expression in rat brain stem.. Am J Physiol.

[12] Yamamoto T, Sawa K (2000). Comparison of c-fos-like immunoreactivity in the brainstem following intraoral and intragastric infusions of chemical solutions in rats.. Brain Res.

[13] Liddle RA, Goldfine ID, Rosen MS, Taplitz RA, Williams JA (1985). Cholecystokinin bioactivity in human plasma. Molecular forms, responses to feeding, and relationship to gallbladder contraction.. J Clin Invest.

[14] Freeman SL, Bohan D, Darcel N, Raybould HE (2006). Luminal glucose sensing in the rat intestine has characteristics of a sodium-glucose cotransporter.. Am J Physiol Gastrointest Liver Physiol.

[15] Hayes MR, Savastano DM, Covasa M (2004). Cholecystokinin-induced satiety is mediated through interdependent cooperation of CCK-A and 5-HT3 receptors.. Physiol Behav.

[16] Andrey P, Maurin Y (2005). Free-D: an integrated environment for three-dimensional reconstruction from serial sections.. J Neurosci Methods.

[17] Burguet J, Andrey P, Rampin O, Maurin Y (2009). Three-dimensional Statistical Modeling of Neuronal Populations: Illustration with Spatial Localization of Supernumerary Neurons in the Locus Coeruleus of Quaking Mutant Mice.. Journal of Comparative Neurology.

[18] Paxinos G, Franklin KBJ (2001). The Mouse Brain In Stereotaxic Coordinates..

[19] Andrey P, Maschino E, Maurin Y (2008). Spatial normalisation of three-dimensional neuroanatomical models using shape registration, averaging, and warping. Fifth IEEE International Symposium on Biomedical Imaging(ISBI'08): From Nano to Macro..

[20] Maschino E, Maurin Y, Andrey P (2006). Joint registration and averaging of multiple 3D anatomical surface models.. Computer Vision and Image Understanding.

[21] Abercrombie M (1946). Estimation of Nuclear Population from Microtome Sections.. The Anatomical Record.

[22] Team RDC (2009). R: A language and environment for statistical computing..

[23] Silverman BW (1986). Density Estimation for Statistics and Data Analysis..

[24] Schwartz GJ (2006). Integrative capacity of the caudal brainstem in the control of food intake.. Philos Trans R Soc Lond B Biol Sci.

[25] Malmierca MS, Izquierdo MA, Cristaudo S, Hernandez O, Perez-Gonzalez D (2008). A discontinuous tonotopic organization in the inferior colliculus of the rat.. J Neurosci.

[26] Luo F, Wang Q, Farid N, Liu X, Yan J (2009). Three-dimensional tonotopic organization of the C57 mouse cochlear nucleus.. Hear Res.

[27] Johnson BA, Leon M (2007). Chemotopic odorant coding in a mammalian olfactory system.. J Comp Neurol.

[28] Petersen CC (2003). The barrel cortex–integrating molecular, cellular and systems physiology.. Pflugers Arch.

[29] Zittel TT, De Giorgio R, Sternini C, Raybould HE (1994). Fos protein expression in the nucleus of the solitary tract in response to intestinal nutrients in awake rats.. Brain Res.

[30] Bowen J, Noakes M, Trenerry C, Clifton PM (2006). Energy intake, ghrelin, and cholecystokinin after different carbohydrate and protein preloads in overweight men.. J Clin Endocrinol Metab.

[31] Morens C, Gaudichon C, Metges CC, Fromentin G, Baglieri A (2000). A high-protein meal exceeds anabolic and catabolic capacities in rats adapted to a normal protein diet.. J Nutr.

