# Supplementary figures and images for: Three-Dimensional Macronutrient-Associated Fos Expression Patterns in the Mouse Brainstem
Source: PLoS One. 2010 Feb 1;5(2):e8974. doi: 10.1371/journal.pone.0008974 (PMC2813867; doi:10.1371/journal.pone.0008974)

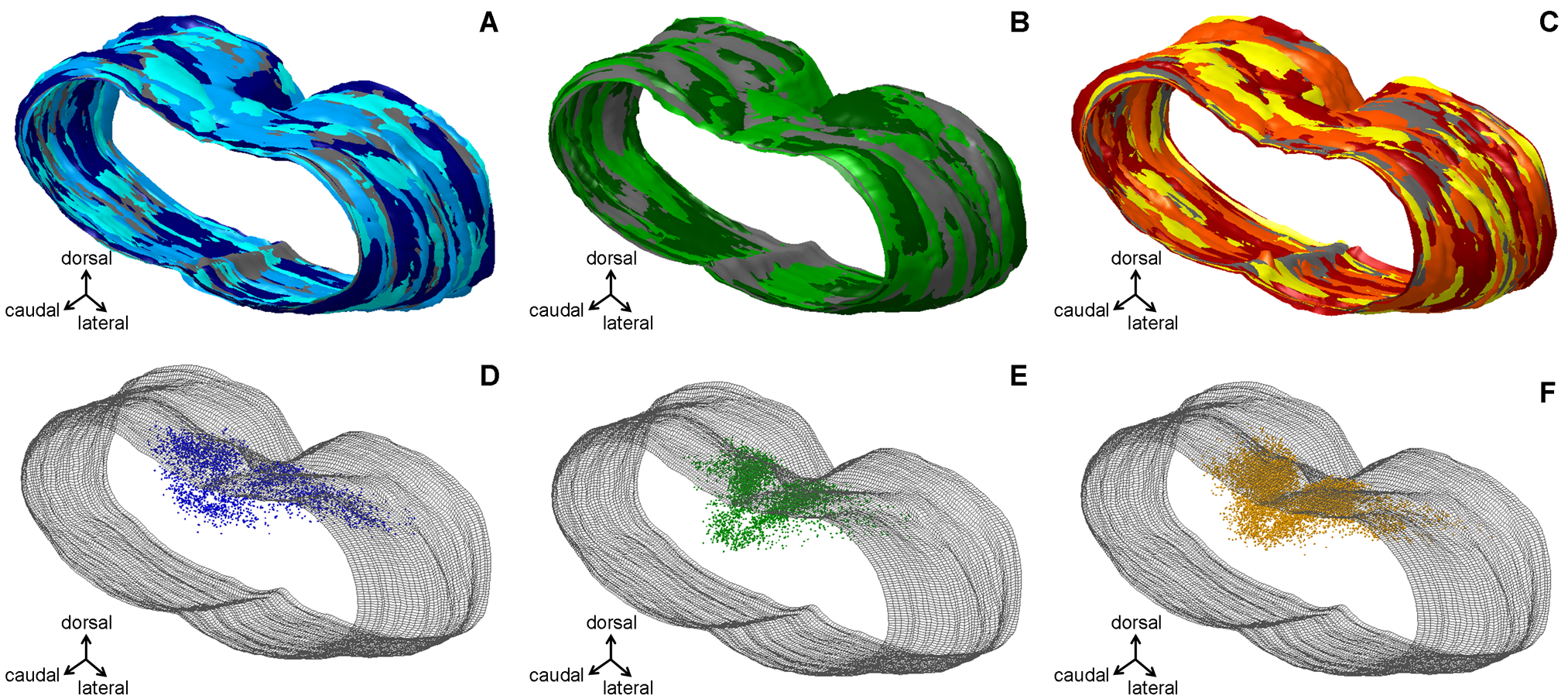

Supplement: Figure S1 — Three-dimensional models of the brainstem and Fos-expressing neurons. A, fusion of the average brainstem model with single brainstem models for mice gavaged with water. B, fusion of the average brainstem model with single brainstem models for mice gavaged with saccharose. C, fusion of the average brainstem model with single brainstem models for mice gavaged with peptone. D, average brainstem model with the total number of neurons marked in mice gavaged with peptone, E, average brainstem model with the total number of neurons marked in mice gavaged with saccharose, F, average brainstem model with the total number of neurons marked in mice gavaged with water. (1.69 MB TIF) [file pone.0008974.s001.tif]

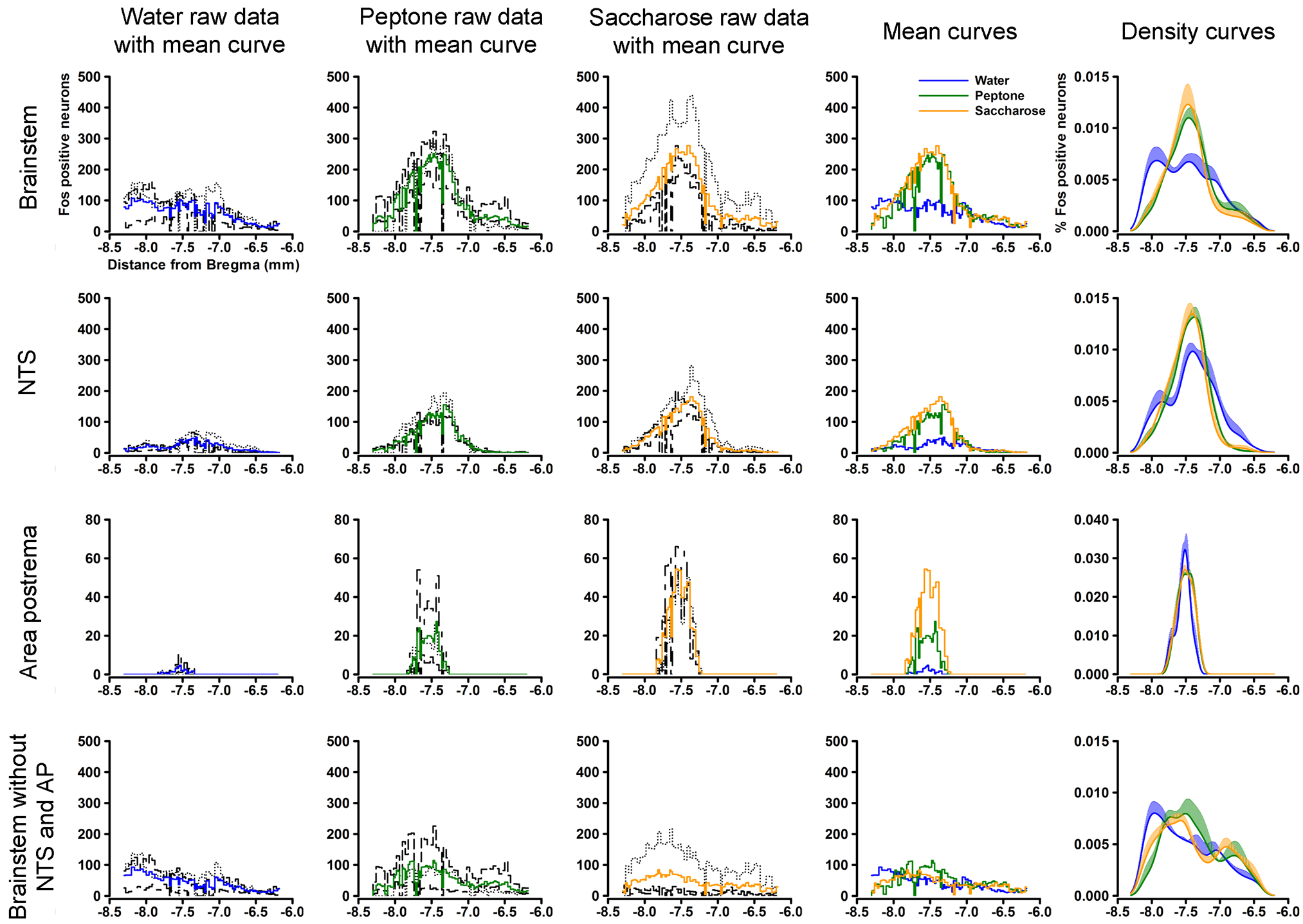

Supplement: Figure S2 — Raw data, means and density curves presenting the distribution of Fos-expressing neurons along the rostrocaudal axis. (0.93 MB TIF) [file pone.0008974.s002.tif]
